# Discriminative Label Relaxed Regression with Adaptive Graph Learning

**DOI:** 10.1155/2020/8852137

**Published:** 2020-12-12

**Authors:** Jingjing Wang, Zhonghua Liu, Wenpeng Lu, Kaibing Zhang

**Affiliations:** ^1^Information Engineering College, Henan University of Science and Technology, Luoyang, China; ^2^School of Computer Science and Technology, Qilu University of Technology (Shandong Academy of Sciences), Jinan, China; ^3^College of Electronics and Information, Xi'an Polytechnic University, Xi'an, China

## Abstract

The traditional label relaxation regression (LRR) algorithm directly fits the original data without considering the local structure information of the data. While the label relaxation regression algorithm of graph regularization takes into account the local geometric information, the performance of the algorithm depends largely on the construction of graph. However, the traditional graph structures have two defects. First of all, it is largely influenced by the parameter values. Second, it relies on the original data when constructing the weight matrix, which usually contains a lot of noise. This makes the constructed graph to be often not optimal, which affects the subsequent work. Therefore, a discriminative label relaxation regression algorithm based on adaptive graph (DLRR_AG) is proposed for feature extraction. DLRR_AG combines manifold learning with label relaxation regression by constructing adaptive weight graph, which can well overcome the problem of label overfitting. Based on a large number of experiments, it can be proved that the proposed method is effective and feasible.

## 1. Introduction

Information technology is developing rapidly and has become a hot topic in recent years. We can get a lot of information from the data, but the dimension of the data is getting higher and higher [1]. On the one hand, the increase of data dimension makes the description of data samples more comprehensive and provides more bases for further analysis and processing of data samples [2]. On the other hand, the increase in the number of features will bring more redundant features, which not only put forward great requirements for hardware and software equipment to complete data processing but also directly affect the reliability and effectiveness of data analysis and processing results [3]. In order to effectively analyze and process the data, by mapping (or transforming) the original data to the low-dimensional space, the features that best reflect the intrinsic nature of the sample data can be obtained. This process is called feature extraction or data dimensionality reduction [4]. The core task of feature extraction or data dimensionality reduction is how to find out which features are most effective for the final data analysis and processing and how to retain the useful information in the data transformation process to the maximum extent. Feature extraction is always a key problem in pattern recognition, which will directly affect the design and performance of the classifier [5–7]. Feature extraction can not only reduce the dimension of data but also retain useful information in the data. It is also widely used in the processing and analysis of complex data.

In recent years, the feature extraction method based on manifold learning has made remarkable achievements in nonlinear data analysis and research and has been widely used in nonlinear data processing and analysis. According to the current popular learning methods, the nonlinear characteristics and manifold structures of the sample data are usually distributed in low-dimensional space [8–11], and the traditional nonlinear subspace method is difficult to describe and extract the information effectively [12–14]. At present, the popular learning method is to obtain the embedded mapping of low-dimensional manifold structure and high-dimensional feature manifold of the sample and then complete the feature extraction of nonlinear information in the sample data [15–18].

Discriminative Fisher embedding dictionary learning (DFEDL) algorithm simultaneously establishes Fisher embedding models on learned atoms and coefficients [19, 20]. In 2000, Tenenbaum from Stanford University published the first manifold learning method based on isometric mapping (ISOMAP) [21] in Science. ISOMAP method is a globally optimized method to preserve the manifold structure between data effectively by maintaining the geometric relationship between sample data (also known as geodesic distance). Roweis proposed a locally linear embedding (LLE) method in literature [22]. By constructing the reconstruction weight between each sample and the neighboring samples, this method can better preserve the manifold features between the neighboring samples when embedding in low-dimensional space [23–26]. Based on Roweis and Tenenbaum's basic research on manifold learning, researchers proposed some improved feature extraction methods, including Laplacian eigenmap (LE) [27], local learning projection (LLP) [28], linear representation-based classifiers (CRC) [29], and linear regression classification (LRC) [30].

Due to the validity of the least-squares regression method in data analysis and the completeness of statistical theory, it is widely used as a basic tool in many machine learning problems including discriminant analysis, clustering, multiview learning, multilabel classification, and semisupervised learning. Sun et al. [31] proposed a least-squares regression model based on generalized eigenvalue decomposition, and Suzanna et al. [32] proposed a weighted least-squares regression model. When solving the multilabel classification problem with least-squares regression, if the data points belong to different categories, then should be considered as the increase of the distance between the data. For example, in order to increase the distance between different types of data points, Leski [33] proposed a quadratic approximation least-square regression model based on misclassification error. However, this model only considers two categories of classification problems. In multilabel classification, the distances among data points from different classes are also expected to be as large as possible [34, 35], as well as in multilabel feature selection. Therefore, multiple least-squares regression models proposed by Leski [33] can be used simultaneously, but the time cost of algorithm implementation will be relatively high.

The traditional label relaxation regression algorithm directly fits the original data without considering the local structure information of the data [36–38]. While the label relaxation regression algorithm for graph regularization considers local geometric information, its performance is largely dependent on graph construction [39, 40]. However, there are two defects in the construction of traditional graphs: first, it is largely influenced by the parameter values; second, it relies on the original data when constructing the weight matrix, which usually contains a lot of noise [41]. This makes the constructed graph often not to be optimal, which affects the subsequent work. To guarantee the global optima of the latent representation and graphs of all views, we integrate the graph completion and common representation learning into a joint optimization framework. [42–44]. Therefore, it is proposed to combine manifold learning with label relaxation regression and construct weight graph through adaptive method. At the same time, local identification information is added on the basis of original linear identification analysis so that the projection learning can grasp the local identification information to expand the identification of the projection. A label relaxation regression algorithm for image classification and feature extraction based on adaptive graph is proposed. In this paper, the main innovation points are as follows:

1. The adaptive graph construction can rightly capture the local structure information of the data
2. The problem of overfitting is avoided by introducing adaptive graph into the objective function of label relaxation regression
3. In order to take full advantage of discriminant information, the global discriminant information based on the LDA is considered

The rest of this paper is arranged as follows: Section 2 briefly reviews DLSR and structured optimal graph. In Section 3, discriminative label relaxed regression with adaptive graph learning (DLRR_AG) is described in detail, and the convergence of the proposed algorithm is proved.  Section 4 mainly provides a lot of experiments to verify the performance of the proposed algorithm. The last Section gives the conclusion.

## 2. Related Work

### 2.1. Discriminative Least-Squares Regression (DLSR) Algorithm

The classification training samples are given *N* numbers, and these samples {(*x*_*i*_, *y*_*i*_)}_*i*=1_^*N*^ fall into *C*(*C* ≥ 2) classes, where *y*_*i*_ ∈ {1,2 …, *c*} is the class label of *x*_*i*_, and *x*_*i*_ is a data point in *R*^*m*^. The linear equation can be satisfied as follows:

(1) $$\begin{array}{c} XW+e_{N}t^{T}\approx Y, \end{array}$$

where *X*=[*x*_1_, *x*_2_,…,*x*_*n*_]^*T*^ ∈ *R*^*N*×*m*^ and *Y*=[*f*_*y*1_, *f*_*y*2_,…,*f*_*yN*_]^*T*^ ∈ *R*^*N*×*C*^. For the ith class, *i*=1,2,…, *c*, $f_{i}={\left[\underset{i-1}{\underset{︸}{0,\ldots 0,1,0,\ldots ,0}}\right]}^{\text{T}}\in R^{c}$, *W* is a transformation matrix in *R*^*m*×*c*^, *t* is a translation vector in *R*^*c*^, and *e*_*N*_=[1,1,…, 1] ∈ *R*^*N*^ is a vector with all 1 s.

Each column vector in Y is of a binary regression type, with the target of class jth being “+1” and the target of the rest being “0.” We can drag these binary outputs far away along two opposite directions. That is, with a positive slack variable, we hope the output will become for the sample grouped into “1” and for the sample grouped into “0.” This treatment can help enlarge the distance between the classes and mapping data point.

Let *B* ∈ *R*^*N*×*C*^ be a constant matrix, in which the ith row and jth column element *B*_*ij*_ is defined as

(2) $$\begin{array}{c} B_{ij}=\left\{\begin{array}{cc} +1, & \text{if }y_{i}=j,\\ -1, & \text{otherwise}. \end{array}\right. \end{array}$$

Each element in *B* corresponds to the direction of the drag. Performing the above *ε* drag on each element of Y, matrix *M* ∈ *R*^*N*×*C*^ records these {*ε*}. The residual can be obtained as follows:

(3) $$\begin{array}{c} XW+e_{N}t^{T}-\left(Y+B⊗M\right)=0, \end{array}$$

where ⊗ is the Hadamard matrix product operator.

The objective function of DLSR can be obtained as follows:

(4) $$\begin{array}{c} \underset{W,t,M}{\text{min}}{\left\|XW+e_{N}t^{T}-Y-B⊗M\right\|}_{F}^{2}+\lambda {\left\|W\right\|}_{F}^{2},\\ s.t.\;M\geq 0, \end{array}$$

where *λ* is a positive regularization parameter. By solving the optimization problem of equation (4), the optimal *W* and *t* can be obtained:

(5) $$\begin{array}{c} W={\left(X^{T}HX+\lambda I_{m}\right)}^{-1}X^{T}HR,\\ t=\frac{\left(R^{T}e_{N}-W^{T}X^{T}e_{N}\right)}{N}, \end{array}$$

where *I*_*m*_ is a *m* × *m* identity matrix and *H*=*I*_*N*_ − (1/*N*)*e*_*N*_*e*_*N*_^*T*^, in which *I*_*N*_ is a *N* × *N* identity matrix, and *R*=*Y*+*B* ⊗ *M* ∈ *R*^*N*×*C*^.

### 2.2. Structured Optimal Graph

It is well known that the data in high-dimension space is usually embedded in low-dimensional manifold [40]. It is a key success factor to preserve local manifold structure information for graph-based methods. The local manifold structure is captured by the similarity matrix, which determines the ultimate performance of graph-based methods.

Suppose that there are *N* training samples from *c* classes which are denoted by *X*=[*x*_1_, *x*_2_,…, *x*_*N*_] ∈ *R*^*N*×*m*^, where *N* is the number of samples used for classifier training, while all N samples are used for determination of S, where *m* is the dimension of observed data, and *x*_*i*_=(*i*=1,2,…, *N*) ∈ *R*^*m*×1^ is the i-th sample for sample set *X*. For any sample *x*_*i*_, it can be connected by all other samples with probability *s*_*ij*_, where *s*_*ij*_ is an element of similarity matrix *S* ∈ *R*^*N*×*N*^. If the distance of two samples is closer, the greater their probability (or similarity) will be, and vice versa. Therefore, the similarity *s*_*ij*_ between *x*_*i*_ and *x*_*j*_ is inversely proportional to their distance. The similarity *s*_*ij*_ can be obtained by solving the following equation:

(6) $$\begin{array}{c} \text{min}\sum_{i,j}\left({\left\|x_{i}-x_{j}\right\|}_{2}^{2}S_{ij}+\alpha S_{ij}^{2}\right),\\ s.t.\;\forall i,s_{i}^{T}1=1,0\leq s_{ij}\leq 1, \end{array}$$

where *S*_*i*_ ∈ *R*^*N*×1^ is a vector whose jth element is *s*_*ij*_ in similarity matrix *S* and *α* is a regularization parameter. The second item in equation (6) is mainly to avoid trivial solutions.

It is the ideal state for each sample to include *c*-nearest-neighbor numbers. That is to say, each *S*_*ij*_(*i*=1,2,…, *N*) in similarity matrix *S* has exact *c* connected components. In fact, the obtained similarity matrix *S* in equation (6) does not meet this requirement in most cases. The problem can be solved as follows. The spectral analysis has an important equation as follows:

(7) $$\begin{array}{c} \sum_{i,j}{\left\|f_{i}-f_{j}\right\|}_{2}^{2}S_{ij}=2Tr\left(F^{T}L_{S}F\right), \end{array}$$

where *F*=[*f*_1_, *f*_2_,…, *f*_*N*_] ∈ *R*^*N*×*C*^ is a class label matrix corresponding to the observed data *X*, *L*_*S*_=*D* − (*S*^*T*^+*S*)/2 is the Laplacian matrix, and matrix *D* is a diagonal matrix whose ith entry is ∑_*j*_*S*_*ij*_+*S*_*ij*_/2.

If the rank of Laplacian matrix *L*_*S*_ equals to *N* − *C*, namely, rank(*L*_*S*_)=*N* − *C*, the obtained similarity matrix *S* will include exact *C* connected components [45]. By combining the constraint to equation (6), equation (6) is written as

(8) $$\begin{array}{c} \text{min}\sum_{i,j}\left({\left\|x_{i}-x_{j}\right\|}_{2}^{2}S_{ij}+\alpha S_{ij}^{2}\right). \end{array}$$

In order to solve equation (6), the ith smallest eigenvalue of Laplacian matrix *L*_*S*_ is denoted by *σ*_*i*_(*L*_*S*_). It is well-known that the solutions of positive semidefinite matrix are more than zero. Laplacian matrix *L*_*S*_ is positive semidefinite, so *σ*_*i*_(*L*_*S*_) ≥ 0. Based on rank(*L*_*S*_)=*N* − *C*, ∑_*i*=1_^*C*^*σi*(*L*_*s*_)=0 is satisfied [45]. According to KyFan's Theorem [46], we have

(9) $$\begin{array}{c} \sum_{i=1}^{C}\sigma i\left(L_{s}\right)=\underset{F^{T}F=I}{\text{min}}Tr\left(F^{T}L_{S}F\right). \end{array}$$

Therefore, based on equation (9), equation (8) can be rewritten as

(10) $$\begin{array}{c} \text{min}\sum_{i.j}\left({\left\|x_{i}-x_{j}\right\|}_{2}^{2}s_{ij}+\alpha s_{ij}^{2}\right)+2\lambda Tr\left(F^{T}L_{S}F\right),\\ s.t.\;\forall i,s_{i}^{T}1=1,0\leq s_{ij}\leq 1,\;F^{T}F=I. \end{array}$$

## 3. Discriminative Label Relaxation Regression Algorithm Based on Adaptive Graph (DLRR_AG)

In this section, the motivation of our DLRR_AG is firstly introduced. Then, the optimum solution of DLRR_AG is given.

### 3.1. The Motivation of DLRR_AG

Traditional label relaxation regression algorithms directly fit the original data, which often results in overfitting. In general, to overcome overfitting, a regularization term is added to the target equation, and a regularization factor is used to balance the target equation and regularization term. In addition to this one, maintaining the information of local manifold structure plays a very important role in improving classification or clustering. There are two kinds of classical nearest-neighbor graphs, one is k-nearest-neighbor graph, and the other is *ε*-nearest-neighbor graph. A large part of graph-based algorithms rely on these two methods to preserve local manifold structure information. However, two problems often occur when these two methods are used. First, the performance of graph learning is greatly affected by the parameter *k* or *ε*, the results of taking different parameter values are sometimes far apart, and the optimal value is not easy to determine, which requires a lot of experiments to obtain, which consumes a lot of time. Second, the traditional graph construction method requires two complicated steps: first, the corresponding weighted matrix should be constructed in adjacent graphs, and then the relaxation regression should be carried out. However, once the weighted matrix is generated from the most primitive observation data, it will not change any more. There is no flexibility. This kind of weighted matrix generated in advance is not practical in practical application, because the original observation data often contain a lot of errors, which will lead to the destruction of the local manifold structure. To solve this problem, we propose a relaxation regression algorithm for adaptive graph. The objective function of the algorithm is described as follows:

(11) $$\begin{array}{c} \underset{W,M}{\text{min}}{\left\|XW-Y-B⊗M\right\|}_{F}^{2}+\sum_{i.j}\left({\left\|W^{T}x_{i}-W^{T}x_{j}\right\|}_{2}^{2}s_{ij}+\alpha s_{ij}^{2}\right)+2\lambda Tr\left(F^{T}L_{S}F\right),\\ s.t.\;M\geq 0,\forall i,s_{i}^{T}1=1,0\leq s_{ij}\leq 1,F^{T}F=I. \end{array}$$

In order to take advantage of intraclass discriminative information, the discriminant information based on the LDA is introduced into the objective function. Equation (11) can be rewritten as

(12) $$\begin{array}{c} \underset{W,M}{\min}{\left\|XW-Y-B⊗M\right\|}_{F}^{2}+\sum_{i.j}\left({\left\|W^{T}x_{i}-W^{T}x_{j}\right\|}_{2}^{2}s_{ij}+\alpha s_{ij}^{2}\right)+2\lambda Tr\left(\;\right.F^{T}L_{S}F\left.\;\right)+\gamma \left(\;\right.Tr\left(\;\right.W^{T}\left(\;\right.S_{w}-S_{b}\left.\;\right)W\left.\;\right),\\ s.t.\;M\geq 0,\forall i,s_{i}^{T}1=1,0\leq s_{ij}\leq 1,F^{T}F=I, \end{array}$$

where *S*_*w*_ and *S*_*b*_ are the within-class scatter matrix and between-class scatter matrix, respectively.

### 3.2. Optimization of DLRR_AG

Equation (12) of the objective function is convex, so it is difficult to get the global optimal solution. Therefore, we can obtain the local optimal solution through continuous iteration. Because the target function contains four different variables, optimization solution (12) is not directly available; it requires an iterative solution to this problem (12). We propose an iterative algorithm to update the rules to solve these problems.

#### 3.2.1. Fixing W, M, and F to Update S

When other variables are fixed, except *S*, equation (12) can be transformed into

(13) $$\begin{array}{c} \text{min}\sum_{i.j}\left({\left\|W^{T}x_{i}-W^{T}x_{j}\right\|}_{2}^{2}s_{ij}+\alpha s_{ij}^{2}\right)+2\lambda Tr\left(F^{T}L_{S}F\right),\\ s.t.\;\forall i,s_{i}^{T}1=1,0\leq s_{ij}\leq 1. \end{array}$$

According to equation (7), equation (13) can be rewritten as

(14) $$\begin{array}{c} \text{min}\sum_{i.j}\left({\left\|W^{T}x_{i}-W^{T}x_{j}\right\|}_{2}^{2}s_{ij}+\alpha s_{ij}^{2}\right)+\lambda \sum_{i,j}{\left\|f_{i}-f_{j}\right\|}_{2}^{2}S_{ij},\\ s.t.\;\forall i,s_{i}^{T}1=1,0\leq s_{ij}\leq 1. \end{array}$$

Because it is independent for the similarity vector of each data point, we can solve the optimal problem for each sample as follows:

(15) $$\begin{array}{c} \text{min}\sum_{i.j}\left({\left\|W^{T}x_{i}-W^{T}x_{j}\right\|}_{2}^{2}s_{ij}+\alpha s_{ij}^{2}\right)+\lambda \sum_{i,j}{\left\|f_{i}-f_{j}\right\|}_{2}^{2}S_{ij},\\ s.t.\;\forall i,s_{i}^{T}1=1,0\leq s_{ij}\leq 1. \end{array}$$

Let *m*_*ij*_=‖*W*^*T*^*x*_*i*_ − *W*^*T*^*x*_*j*_‖_2_^2^, *n*_*ij*_=‖*f*_*i*_ − *f*_*j*_‖_2_^2^, and *d*_*ij*_=*|m*_*ij*_+*λn*_*ij*_; equation (15) can be rewritten as

(16) $$\begin{array}{c} \text{min}{\left\|s_{i}+\frac{1}{2\alpha }d_{i}\right\|}_{2}^{2},\\ s.t.\;s_{i}^{T}1=1,\;0\leq S_{ij}\leq 1. \end{array}$$

#### 3.2.2. Fixing S, W, and M to Update F

When other variables are fixed, except *F*, equation (12) can be transformed into

(17) $$\begin{array}{c} \text{min}Tr\left(F^{T}L_{S}F\right),\\ s.t.\;F^{T}F=I. \end{array}$$

The optimal solution of *F* is formed from the eigenvectors of the *c* minimum eigenvalues in the *L*_*S*_.

#### 3.2.3. Fixing S, W, and F to Update M

When other variables are fixed, except *M*, equation (12) can be transformed into

(18) $$\begin{array}{c} \text{min}{\left\|XW-Y-B⊗M\right\|}_{2}^{2},\\ s.t.\;M\geq 0. \end{array}$$

Let us now consider optimization in terms of *M* ∈ *R*^*N*×*C*^. Given *W* and *T*, and let *P*=*XW* − *Y* record the regression error of *n* data points, then the optimization problem can be solved from the following aspects:

(19) $$\begin{array}{c} \text{min}{\left\|P-B⊗M\right\|}_{F}^{2},\\ s.t.\;M\geq 0. \end{array}$$

According to the square of matrix Frobenius norm, the fact that an element can be decoupled, (10) can be decoupled equivalently into *n* × *c* subproblems. For the ith row of the matrix and the jth column element *M*_*ij*_, we have

(20) $$\begin{array}{c} \underset{{\text{M}}_{ij}}{\text{min}}{\left\|P_{ij}-B_{ij}{\text{M}}_{ij}\right\|}_{F}^{2},\\ s.t.\;M\geq 0, \end{array}$$

where *P*_*ij*_ and *B*_*ij*_ are the ith row and jth elements of *P* and *B*, respectively.

Note that *B*_*ij*_^2^=1. Thus, we have (*P*_*ij*_ − *B*_*ij*_M_*ij*_)^2^=(*B*_*ij*_ − *P*_*ij*_M_*ij*_)^2^. Then, the optimization problem of equation (20) can be rewritten as

(21) $$\begin{array}{c} \underset{{\text{M}}_{ij}}{\text{min}}{\left(B_{ij}P_{ij}-{\text{M}}_{ij}\right)}^{2},\\ s.t.\;M_{ij}\geq 0. \end{array}$$

Obviously, the optimal solution of equation (21) is given as follows:

(22) $$\begin{array}{c} M=\text{max}\left(B⊗P,0\right). \end{array}$$

#### 3.2.4. Fixing S, M, and F to Update W

Equation (12) is a constrained convex optimization problem. According to the properties of convex optimization, the local optimal solution is also the optimal solution of the whole. Next, this paper uses the iterative method to solve the optimal solution of equation (12).

To solve W, given M, equation (12) is an unconstrained convex optimization problem for W. You just take the derivative of that and you set the derivative to 0 and you get W.

When other variables fix, except *W*, equation (12) can be transformed into

(23) $$\begin{array}{c} \underset{W}{\text{min}}{\left\|XW-Y-B⊗M\right\|}_{F}^{2}+\sum_{i.j}{\left\|W^{T}x_{i}-W^{T}x_{j}\right\|}_{2}^{2}+\gamma \left(tr\left(W^{T}\left(S_{w}-S_{b}\right)\right)W\right). \end{array}$$

Let *N*=*Y*+*B* ⊗ *M*, then the problem in (23) can be transformed into the following problem:

(24) $$\begin{array}{c} \underset{W}{\text{min}}{\left\|XW-N\right\|}_{F}^{2}+\lambda Tr\left(W^{T}XLX^{T}W\right)+\gamma \left(tr\left(W^{T}\left(S_{w}-S_{b}\right)W\right)\right)\\ =Tr\left(W^{T}X^{T}XW-W^{T}X^{T}N\right)+\lambda Tr\left(W^{T}XLX^{T}W\right)+\gamma \left(tr\left(W^{T}\left(S_{w}-S_{b}\right)W\right)\right)\\ =Tr\left(W^{T}\left(X^{T}X+\lambda XLX^{T}+\gamma \left(S_{w}-S_{b}\right)\right)W-W^{T}X^{T}N\right)\\ \Rightarrow W={\left(X^{T}X+\lambda XLX^{T}+\gamma \left(S_{w}-S_{b}\right)\right)}^{-1}X^{T}N, \end{array}$$

where *L*=*D* − *S* is the graph Laplacian and *D* is a diagonal matrix whose diagonal elements *D*_*ii*_=∑_*j*_*S*_*ij*_.

## 4. Experiments

In this section, we perform a large number of experiments, and the experimental results can prove that our proposed DLRR_AG algorithm can achieve high classification accuracy. In order to prove the effectiveness of our DLRR_AG algorithm, six public image databases are used to validate our method. For the sake of contrast, the proposed DLRR_AG algorithm is compared with other typical feature extraction methods as follows:

- Collaborative representation-based classification (CRC) [29]: CRC is a combined result of machine learning and compressed sensing, which shows its good classification performance on face image data. Considering the training samples from a specific class and the query set as two linear subspaces, the classwise prototypes most correlated with the query set are learned, resulting in a condensed gallery set.
- Linear regression classification (LRC) [30]: LRC has attracted a great amount of attention owning to its promising performance in face recognition. However, its performance will dramatically decline in the scenario of limited training samples per class, particularly when only single training sample is available for a specific person.
- Flexible manifold embedding (FME) [45]: FME is a semisupervised manifold learning framework with good applicability. It can effectively utilize label information from labeled data as well as a manifold structure from both labeled and unlabeled data.
- Joint global and local structure discriminant analysis (JGLDA) [46]: for linear dimension reduction, it preserves the local intrinsic structure, which characterizes the geometric properties of similarity and diversity of data by two quadratic functions.
- Flexible linear regression classification (FLRC) [47]: the inferences are based on the least-squares estimators of the model which have been shown to be coherent with the interval arithmetic defining the model and to verify good statistical properties.
- Discriminative least-squares regression (DLSR) [34]: DLSR is to embed class label information into the LSR formulation such that the distances between classes can be enlarged. In order to implement this idea, a technique called *ε*-dragging is introduced to force the regression targets of different classes moving along with opposite directions.

### 4.1. Experiments on YALE Database

**Figure 1:**
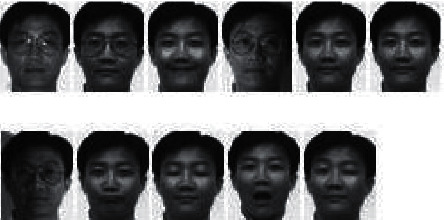
Sample face images from Yale database.

Yale faces a database of 15 people containing 165 photos. They were taken by 15 people under different lighting conditions and with different facial expressions. Each person took 11 pictures. In our experiment, each image was manually cropped to a size of 50 by 40 pixels.  Figure 1 shows the sample images of one of them.

In this experiment, the first 2, 3, to 6 images from each object are used for training set, and the rest is utilized for test set. In order to evaluate the algorithm more objectively, we will eliminate random effects on the algorithm in the process of implementation, and all the methods are repeated 10 times. The recognition rates of all algorithms are shown as Table 1.

It can be seen from Table 1 that we can draw two points. Firstly, the recognition performance of the proposed DLRR_AG method is better than DLSR method irrespective of the number of training samples. Secondly, our method is superior to all other methods, except when the training sample is 4.

### 4.2. Experiments on ORL Database

**Figure 2:**
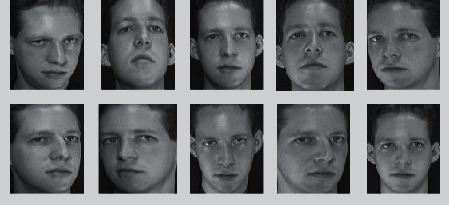
Sample face images from ORL face database.

The ORL dataset includes 400 face images from 40 different objects, and each object has 10 face images. For some people, their images were taken at different time and different light; image content includes different facial expression and facial details.  Figure 2 shows the sample images of one of them.

In this experiment, the training set was the first 3, 4, 5, and 6 images of each person, and the test set was the remaining images. All algorithms are repeated 10 times. The recognition rates of each algorithm are shown in Table 2.

We can clearly see from Table 2 that the proposed DLRR_AG is superior to CRC, LRC, FLRC, FME, LRR JGLDA, and DLSR.

### 4.3. Experiments on Georgia Tech Database

**Figure 3:**
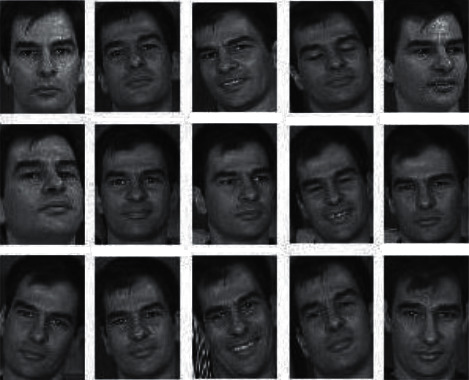
Sample face images from Georgia Tech face database.

The Georgia Tech face database contains photos of 50 people taken during two or three sessions and produced at Georgia Tech. In the database, each individual took 15 color JPEG images with a cluttered background and a resolution of 640 by 480 pixels. The faces in these pictures may be front and tilted, or they may be front or tilted. These images include the different expressions, illuminations, and proportions. Each image is manually cropped to 60 by 50 pixels. All images are converted to grayscale images in the experiment.  Figure 3 shows the sample images of one of them.

In this experiment, the training set was the first 4, 5, to 8 images of each person, and the test set was the remaining images. Repeat the algorithm 10 times. The recognition rates of each algorithm are shown in Table 3.

As can be seen from Table 3, DLRR_AG performs well compared with all other algorithms on the Georgia Tech database. In particular, the performance of DLRR_AG is much higher than that of CRC.

### 4.4. Experiments on CMU PIE Database

**Figure 4:**
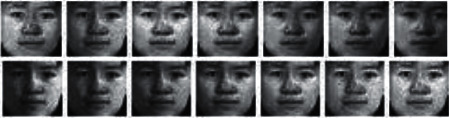
Some face images of one object from CMU PIE database.

The CMU PIE database contains 41,368 facial images. The images were taken by 68 people with different expressions, decorations, and postures. The acquisition of multiple images of each object is based on the premise of fixed expression and attitude, and the illumination is changed to obtain 14 face images, and then these images are cut to 32 × 32 pixels.  Figure 4 shows multiple images from an object.

In this experiment, the first 4, 5, 6, 7, and 8 face images of each object are selected to act as training set, and the rest are taken as test set. The proposed algorithms are repeated for 10 times. The recognition rates of all algorithms are shown in Table 4.

From Table 4, we can know that the proposed DLRR_AG can respectively obtain the best recognition performance in the corresponding comparison algorithms irrespective of the variations of training sample size.

### 4.5. Experiments on AR Database

**Figure 5:**
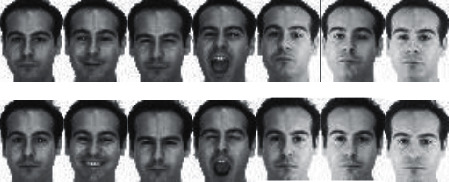
Sample face images from AR database.

The AR face database [44] contains color face images of 120 people, and the total number of face images exceeds 4,000. Among them, 120 subjects were photographed twice with different facial expressions, light conditions, and shade, with a 14-day interval, and each person produced 26 images. In our experiment, out of the 26 facial images of 120 people, seven were selected from each stage, or 14 face images per person. Each image is manually cropped to 50 by 40 pixels. All images are converted to grayscale images.  Figure 5 shows the sample images from one object.

In this experiment, we obtained 14 images of unmasked faces from the first and second experiments. The images in the first stage (ranging from 3 to 7) were used as training images, and the face images in the second stage were used as test images. The proposed algorithms are repeated for 10 times. The experiment results are shown in Table 5.

As can be seen from Table 5, compared with the proposed DLRR_AG algorithm, the classification results of CRC, LRC, FLRC, FME LRR, and JGLDA are poor. In other words, the proposed method can achieve the best recognition performance.

### 4.6. Experiments on UMIST Database

**Figure 6:**
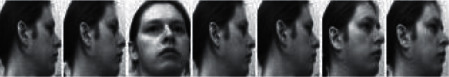
Sample face images from the UMIST database.

The UMIST face database contains a total of 575 facial images of 20 people. The 575 photos had all the poses of 20 people in the database, a mix of race, gender, and physical appearance. The views are different for each topic, between 19 and 48 views for each topic. The size of the face image in the view is 56 by 48 pixels.  Figure 6 shows example images of a person.

In this experiment, the first 1, 2 to 5 face images of each person are generally selected as the training set, while other images are used as the test images. The algorithm is repeated 10 times for each test. The experimental results are shown in Table 6.

As can be seen from Table 6, when the training sample size is 3, the recognition rate of DLRR_AG algorithm is slightly lower than that of DLSR algorithm. However, the recognition rate of the proposed DLRR_AG algorithm is higher than other algorithms when the training sample size is not 3.

## 5. Conclusion

This paper presents a discriminative label relaxation regression algorithm based on adaptive graph (DLRR_AG) algorithm, which can effectively alleviate the overfitting problem caused by label relaxation by correctly capturing the local structure information of the original observed data. The main innovation of this paper has the following points. (1) The adaptive neighborhood graph can well capture the essential local structural information of the original data. (2) Label relaxation, manifold learning, and discriminant analysis are integrated into a unified framework. A large number of experiments in six public image databases show that the proposed method is superior to other related methods. Therefore, the proposed method is effective and feasible.

## Figures and Tables

**Table 1:** Average recognition rate of different methods on the Yale database (%).

Methods	The number (or proportion) of the training samples per class				
	2 (18.18%)	3 (27.27%)	4 (36.36%)	5 (45.45%)	6 (54.54%)
CRC	67.14	66.79	65.83	70.06	74.52
LRC	65.73	62.94	61.89	69.76	73.79
FLRC	66.17	65.98	67.85	70.85	74.85
FME	60.73	62.94	**70.89**	71.86	76.34
JGLDA	68.55	69.45	69.64	75.48	75.82
DLSR	67.38	67.62	66.07	70.60	75.48
DLRR_AG	**69.17**	**70.71**	70.60	**73.10**	**76.55**

Bold values indicate the highest recognition rate of all methods.

**Table 2:** Average recognition rate of different methods on the ORL database (%).

Methods	The number (or proportion) of the training samples per class			
	3 (30%)	4 (40%)	5 (50%)	6 (60%)
CRC	87.86	91.25	92.00	93.75
LRC	82.14	85.42	89.50	95.60
FLRC	86.56	88.50	93.20	95.61
FME	83.00	86.42	90.12	95.33
JGLDA	88.55	89.63	92.93	93.61
DLSR	88.45	90.23	92.66	93.25
DLRR_AG	**88.93**	**91.67**	**94.50**	**95.63**

Bold values indicate the highest recognition rate of all methods.

**Table 3:** Average recognition rate of different methods on the Georgia Tech database (%).

Methods	The number (or proportion) of the training samples per class				
	4 (26.67%)	5 (33.33%)	6 (40.00%)	7 (46.67%)	8 (53.33%)
CRC	57.64	61.43	68.11	71.25	75.40
LRC	56.36	59.60	66.67	70.00	74.57
FLRC	58.22	60.18	67.10	71.38	75.20
FME	53.28	58.56	67.55	70.22	73.55
JGLDA	57.68	59.32	66.79	68.95	72.84
DLSR	50.55	54.40	65.33	69.88	72.10
DLRR_AG	59.27	63.60	68.22	73.00	75.42

**Table 4:** Average recognition rate of different methods on the CMU PIE database (%).

Methods	The number (or proportion) of the training samples per class				
	4 (19.05%)	5 (23.81%)	6 (28.57%)	7 (33.33%)	8 (38.10%)
CRC	79.43	84.27	87.68	88.99	92.20
LRC	70.72	79.61	82.86	87.57	89.62
FLRC	78.22	80.23	84.36	88.45	91.33
FME	70.93	77.52	81.33	85.07	86.96
JGLDA	78.55	79.78	79.95	88.63	88.69
DLSR	74.25	80.29	85.98	88.74	91.95
DLRR_AG	**81.50**	**84.71**	**88.15**	**89.75**	**92.59**

Bold values indicate the highest recognition rate of all methods.

**Table 5:** Average recognition rate of different methods on the AR database (%).

Methods	The number (or proportion) of the training samples per class				
	3 (21.43%)	4 (28.57%)	5 (35.71%)	6 (42.86%)	7 (50.00%)
CRC	70.71	70.60	73.10	76.55	77.38
LRC	66.79	65.83	70.06	74.52	75.12
FLRC	67.62	66.07	70.60	75.48	75.71
FME	70.88	70.96	73.30	76.85	77.84
JGLDA	68.55	69.45	69.64	75.48	75.82
DLSR	67.62	66.07	70.60	75.48	75.71
DLRR_AG	**71.50**	**72.19**	**74.67**	**77.29**	**79.31**

Bold values indicate the highest recognition rate of all methods.

**Table 6:** Average recognition rate of different methods on the UMIST database (%).

Methods	The number (or proportion) of the training samples per class				
	1 (21.05%)	2 (26.31%)	3 (31.58%)	4 (36.84%)	5 (42.10%)
CRC	46.77	60.00	71.55	74.68	84.33
LRC	48.53	60.56	71.72	75.63	84.60
FLRC	49.93	60.88	72.87	76.24	83.97
FME	56.25	58.28	69.79	75.31	83.29
JGLDA	53.13	59.67	69.66	78.59	87.80
DLSR	55.45	60.00	**74.25**	80.23	85.98
DLRR_AG	**56.78**	**61.69**	73.79	**81.61**	**88.28**

Bold values indicate the highest recognition rate of all methods.

## Data Availability

The image databases used in this paper are publicly available for scientific research.
